# The incidence and drug resistance of *Clostridium difficile* infection in Mainland China: a systematic review and meta-analysis

**DOI:** 10.1038/srep37865

**Published:** 2016-11-29

**Authors:** Chenjie Tang, Lunbiao Cui, Yuqiao Xu, Le Xie, Pengfei Sun, Chengcheng Liu, Wenying Xia, Genyan Liu

**Affiliations:** 1 Department of Laboratory Medicine, The First Affiliated Hospital of Nanjing Medical University, Nanjing 210029, P.R. China; 2 Key Laboratory of Enteric Pathogenic Microbiology of Ministry of Health, Jiangsu Provincial Center for Disease Control and Prevention, Nanjing 210009, P.R. China

## Abstract

It has been widely reported that the incidence and severity of *Clostridium difficile* infection (CDI) have increased dramatically in North America and Europe. However, little is known about CDI in Mainland China. In this study, we aimed to investigate the incidence of CDI and the main epidemic and drug-resistant strains of *C. difficile* in Mainland China through meta-analysis of related studies published after the year 2010. A total of 51 eligible studies were included. The pooled incidence of toxigenic *C. difficile* among patients with diarrhoea was 14% (95% CI = 12–16%). In Mainland China, ST-37 and ST-3 were the most prevalent strains; fortunately, hypervirulent strains, such as ST-1 (BI/NAP1/027) and ST-11 (RT 078), have only occurred sporadically to date. The rates of *C. difficile* resistance to ciprofloxacin (98.3%; 95% CI = 96.9–99.7%), clindamycin (81.7%; 95% CI = 76.1–87.3%) and erythromycin (80.2%; 95% CI = 73.5–86.9%) are higher than in other counties; however, none of the *C. difficile* isolates reported in Mainland China were resistant to metronidazole (n/N = 0/960), vancomycin (n/N = 0/960), tigecycline (n/N = 0/41) or piperacillin/tazobactam(n/N = 0/288).

*Clostridium difficile (C. difficile*) is a Gram-positive, spore-forming, anaerobic bacterium that can colonize the human colon. Antibiotic therapy can have the adverse effect of disrupting the normal flora of the gut, in which case *C. difficile* may opportunistically dominate the gut and induce colitis. Not until J. G. Bartlett discovered the relationship between *C. difficile* and pseudomembranous colitis (PMC) in 1978^1^ was attention focused on this anaerobic bacteria^2^. Indeed, Bartlett found that almost all PMC cases are caused by *C. difficile*^3^.

*C. difficile* produces toxins, including enterotoxin (toxin A, *tcd*A) and cytotoxin (toxin B, *tcd*B). Although the pathogenicity of toxin A is still debated, the function of toxin B in disease is well acknowledged in infected patients. Some strains of *C. difficile*, like RT 027, seem to be highly virulent. Some of the base-pairs of the negative regulatory gene *tcdC* are missing in this strain, which increases the production of toxin A and toxin B more than 16- and 23-fold, respectively. This increased pathogenicity is relevant to most outbreaks in Europe and North America^4^. According to several studies from the early 2000s, the incidence and severity of *C. difficile* infection (CDI) have increased dramatically in North America and Europe^5^,^6^,^7^. Another study found that *C. difficile* was the most commonly detected diarrhoeal pathogen in Western Australia^8^, and *C. difficile* RT 027 has been identified as the major strain responsible for outbreaks^9^,^10^,^11^,^12^. Moreover, the incidence and severity of hospital-acquired and community-acquired CDI are increasing worldwide^13^. One investigation published in *The New England Journal of Medicine* in 2014 showed that *C. difficile* was the most commonly reported pathogen (causing 12.1% of all health care–associated infections) in America^14^. Furthermore, *C. difficile* has become the primary pathogenic faecal bacteria associated with hospital infection, and the overall incidence of CDI has exceeded that of methicillin-resistant *Staphylococcus aureus* (MRSA) in parts of the United States^15^,^16^. Although there have been numerous studies of the epidemiology of CDI in North America and Europe, few studies have been undertaken elsewhere, particularly in Asia^17^.

There are many risk factors for CDI, including the use of antibiotics or proton pump inhibitors (PPIs), hospitalization, aging, gastrointestinal surgery, and conditions that may affect the colonic flora, among others^18^,^19^. However, exposure to antibiotics is the most important risk factor for the development of CDI^5^, and a high prevalence of indiscriminate and inappropriate use of antimicrobials exists in Asia^17^. In Mainland China, antibiotics are heavily consumed, and the abuse of antibiotics is fairly serious^20^. The rate of antibiotic usage by inpatients in Mainland China is as high as 80%, and the use of broad-spectrum antibiotics and joint use accounted for 58% of antibiotic treatments before 2004, according to an investigation by the WHO. This rate surpassed the international level (30%) to a large extent^20^,^21^. Another report found that the rate of antibiotic usage by inpatients in Mainland China was approximately 70% according to the Ministry of Health National Antimicrobial Resistance Investigation Net (Mohnarin). Moreover, almost every surgical patient receives antibiotics, and this usage rate is as high as 97%. In addition, the consumption of antibiotics per person each year in Mainland China is approximately 138 g on average, whereas only 13 g of antibiotics are consumed per person per year in the United States^22^. Although CDI is recognized as a major epidemic organism in North America and Europe, Chinese hospitals only sporadically report CDI. No large-scale studies have been performed to date, and therefore the status of CDI in Mainland China remains unknown. Moreover, the recognition of CDI among general healthcare workers and patients in Mainland China is very poor. This study intends to expand the effective research data and provide a more reliable meta-analysis based conclusion on the actual status of CDI and drug resistance in Mainland China. In particular, the following themes were focused and investigated: Topic 1: the incidence of CDI in Mainland China; Topic 2: the molecular epidemiology of *C. difficile* in Mainland China; and Topic 3: the antibiotic resistance of *C. difficile* in Mainland China. This information is critical for developing appropriate strategies to prevent CDI and the vast negative impact of such infections in Mainland China.

## Results

### Search results

**Figure 1:**
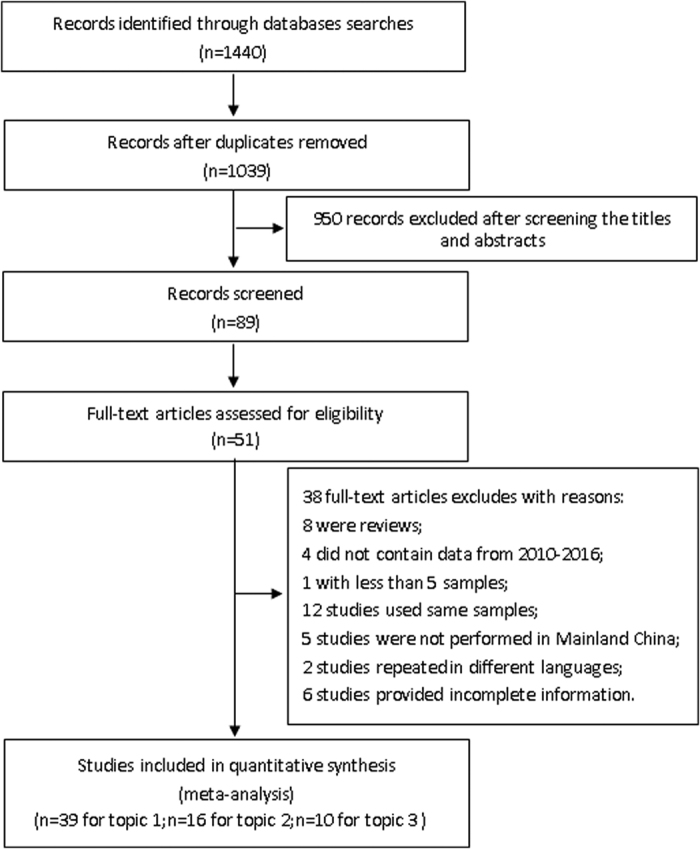
Flow diagram of the procedures used to identify CDI studies in Mainland China.

We reviewed 7 electronic databases and identified 1,440 articles published from 2010 to 2016 (Fig. 1). After initial evaluation of the titles and abstracts, 1,351 articles were excluded because of their irrelevance and duplication. The full text of the remaining articles was reviewed. Among the 89 articles, 38 were excluded again for specific reasons: 8 were reviews, 4 studies did not include data from 2010–2016, 1 study had fewer than 5 samples, 12 studies used the same samples, 5 studies didn’t originate from Mainland China, 2 studies were repeated in different languages, and incomplete information was provided in 6 studies. Finally, 51 studies were included in this systematic review and meta-analysis. Of these, 39 articles focused on Topic 1, 16 focused on Topic 2 and 10 focused on Topic 3.

### Characteristics of the eligible studies

All features of the 51 eligible studies are listed in Supplementary Tables 1, 2 and 3. Regarding Topic 1, Beijing and Guangdong each produced 6 articles; 4 articles were from Hunan; Hubei and Shanghai each produced 3 articles; Jiangsu, Hebei and Sichuan each produced 2 articles; 7 articles concerned Zhejiang; and 1 article each originated from Anhui, Gansu, Henan, and Ningxia. A total of 15,313 samples were collected to detect the existence of *C. difficile* and the associated toxins. Some 16 of the included studies were related to Topic 2, and multi-locus sequence typing (MLST) and PCR ribotyping (RT) were used to detect the molecular type of *C. difficile*. Ten articles were relevant to Topic 3. Antibiotic sensitivity testing (AST) were carried out with 3 methods differently; 6 used the agar dilution method; 3 used the E-test; and 1 study combined the agar dilution, disk diffusion and E-test methods.

### The incidence of CDI in Mainland China

**Figure 2:**
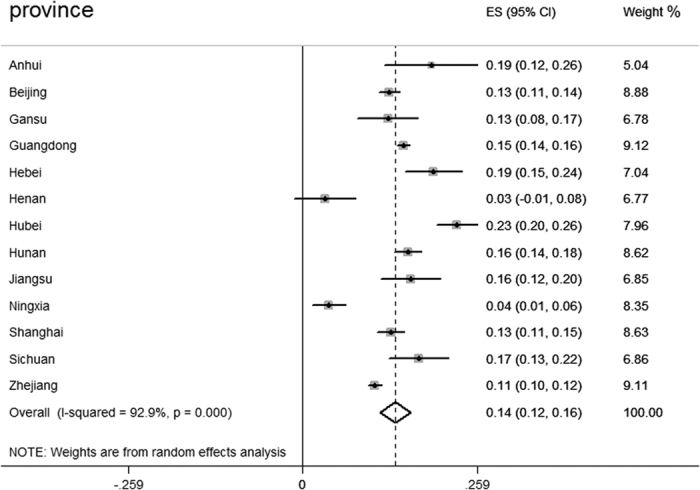
Meta-analyses of CDI in Mainland China.

**Figure 3:**
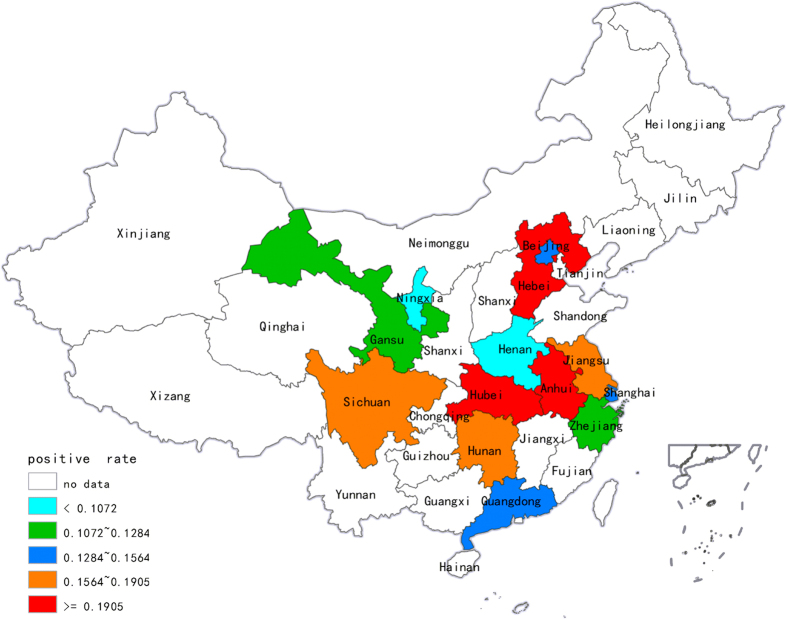
The incidence of CDI in Mainland China (Created by an online Chinese website.http://c.dituhui.com/apps/range).

In total, 39 studies, including 13 provinces and 15,313 samples, reported the positive rate of toxigenic *C. difficile* (Table 1). We estimated the mean positive rate of toxigenic *C. difficile* in diarrhoea patients in Mainland China to be 14% (95% CI = 12–16%) (n/N = 2,132/15,313), with a high level of heterogeneity between the estimated rates (I-squared = 92.9%, p < 0.001) (Fig. 2). Figure 3 shows the incidence of CDI in mainland China over the past 6 years. In particular, the positive rates reported in Hubei (23%; 95% CI = 20–26%), Hebei (19%; 95% CI = 15–24%), Anhui (19%; 95% CI = 12–26%) and Sichuan (17%; 95% CI = 13–22%) were very high, whereas Ningxia (4%; 95% CI = 1–6%) and Henan (3%; 95% CI = −1–8%) showed lower positive rates.

### The molecular epidemiology of *C. difficile*

We next reviewed the molecular classification methods, including MLST and RT, for *C. difficile* in the published articles. As shown in Table 2, ST-37 (17.2%; 95% CI = 12.2–22.1%) (n/N = 152/913) and ST-3 (18.1%; 95% CI = 8.3–27.8%) (n/N = 67/295) were the dominant strains in Mainland China. Focusing on studies that used the RT method, RT 006 (55%; 95% CI = 39.6–70.4%) (n/N = 22/40) was the predominant strain in Mainland China, followed by RT 017 (17.2%; 95% CI = 12.2%1–22.1%) (n/N = 152/913) as the second epidemic strain. Fortunately, hypervirulent strains, such as ST-1 (BI/NAP1/027) and ST-11 (RT 078), were only reported sporadically, and no outbreaks have been reported to date (Table 3).

### The antibiotic resistance of *C. difficile*

A total of 24 drugs were reported in the included studies. However, 14 drugs were investigated by several studies (Table 4), while the others were only examined in single studies. The results shown in Table 4 are more credible because they include several studies and a large number of samples. None of these strains were resistant to metronidazole (n/N = 0/960), vancomycin (n/N = 0/960), piperacillin/tazobactam (n/N = 0/288), or tigecycline (n/N = 0/41). However, these strains were highly resistant to erythromycin (80.2%; 95% CI = 73.5–86.9%) (n/N = 340/433), clindamycin (81.7%; 95% CI = 76.1–87.3%) (n/N = 476/581) and ciprofloxacin (98.3%; 95% CI = 96.9–99.7%) (n/N = 688/694). The results of the other 10 single-drug studies showed that the rate of resistance to teicoplanin was 0% (n/N = 0/20), amoxicillin/clavulanic acid was 0% (n/N = 0/22), ampicillin was 0% (n/N = 0/22), ceftriaxone was 100% (n/N = 20/20), piperacillin was 0% (n/N = 0/22), penicillin was 27.3% (n/N = 6/22), fidaxomicin was 0% (n/N = 0/45), chloramphenicol was 2% (n/N = 7/334), cefoxitin was 92.6% (n/N = 87/94) and imipenem was 3% (n/N = 4/138). None of the strains were resistant to fidaxomicin, amoxicillin/clavulanic acid, ampicillin, piperacillin, or teicoplanin, and nearly all of the strains were sensitive to chloramphenicol and imipenem according to the published articles^23^,^24^,^25^,^26^,^27^,^28^. The strains reported in one article were resistant to ceftriaxone and cefoxitin^25^,^29^.

## Discussion

Because only a limited number of studies have investigated *C. difficile* in Mainland China, some provinces were only investigated in one study and others were not included in the analyses. Although the data were not sufficient to provide an accurate conclusion, we still obtained valuable information through meta-analysis of related published data. In this study, we found that the incidence of toxigenic *C. difficile* in Mainland China was 14% (95% CI = 12–16%) (n/N = 2,132/15,313). Fortunately, highly virulent strains were rarely reported, although CDI outbreaks have been reported more than 100 times in North America and Europe since 2003^30^. From 2000 to 2009, the confirmed CDI cases were more than doubled from 139,000 to 336,600 in US hospitals, and the number of patients with a primary confirmed CDI cases tripled from 33,000 to 111,000^31^. Moreover, the incidence, mortality, and medical care costs of CDIs have reached historic highs in America^31^. However, Asian countries seem to have a low incidence of CDI. One survey conducted in Japan found only a small number of patients with serious CDI and recurrent CDI. Meanwhile, regarding the annual number of CDI patients at all of the hospitals that participated in this survey, the largest group of hospitals reported treating “1 to 5 patients a year,” representing 17.8% of the sample, and the second largest group of hospitals reported treated “no patients a year,” representing 13.1%^32^. In addition, the CDI incidence in Korea was estimated to be 5.06 per 100,000 population in 2011, whereas the estimated incidence in America per 100,000 population ranged from 30 to 120 cases of community-associated infection and from 50 to 160 cases of health care-associated infection across the Emerging Infections Program (EIP) sites in the same year^33^,^34^. In Mainland China, the positive rate of toxigenic *C. difficile* infection in some provinces, such as Ningxia and Henan, was close to zero. This result was different with Jin K *et al*. reviewed in 2010, in which no significant geographical variation of CDI was found in Mainland China^35^. Therefore, we re-analysed the articles concerning Ningxia and Henan. The CDI study in Henan only included 60 samples, and the authors detected 9 cases of *C. difficile*, but only 2 produced toxins. The investigation in Ningxia included 233 diarrhoea patients; of these, 162 cases were due to non-infectious diarrhoea and 71 cases were caused by pathogenic bacteria, where 9 *C. difficile* strains were assayed with the VIDAS CDAB kit. On the other hand, Anhui, Hebei, Hubei and Sichuan reported a much higher positive rate, which attracted our attention. These studies were reviewed, which showed that some risk factors were associated with these results. First, the diarrhoea patients included in these studies represented special groups; some diarrhoea patients came from the intensive care unit (ICU), some suffered from hematologic diseases, and some patients had used antibiotics for a long time. Moreover, some diarrhoea patients were diagnosed with antibiotic-associated diarrhoea (AAD). Thus, these results may increase the positive rates of CDI to a high level. In addition, 2,570 patients from 9 articles were diagnosed with AAD or confirmed to have used antibiotics, and this feature undoubtedly increased the rate of CDI in our survey. Notably, studies from Guangdong province included one-third of all the pooled nationwide cases approximately, which may have impacted the overall results of this study. Thus, reanalysis was necessary. We deleted the data from Guangdong and recalculated the mean incidence of CDI based on the data from the other 12 provinces. Finally, the recalculated incidence of CDI was 14% (95% CI = 11–16%) (n/N = 1,343/10,024). The same phenomenon was found from the studies of Zhejiang province. Therefore, we recalculated without the data from Zhejiang and got the same result of 14% (95% CI = 12–17%) (n/N = 1,732/11,583). Therefore, these changes did not produce a significant difference in the overall rate of CDI, in other words, the data from Guangdong and Zhejiang had a small impact on the nationwide average value of CDI incidence despite their big data weight.

Based on the risk factors mentioned above, some researchers are worried about the incidence of CDI in Mainland China due to the growing elderly population and the well-recognized problem of over-prescribing broad-spectrum antibiotics in Mainland China^35^. The lifestyles of Chinese people, particularly those of young Chinese, have changed tremendously over the last 20 years, partially because of the rapid growth of the Chinese economy. These lifestyle changes, which were not as common in Mainland China in the past, may represent a major contributor to the increasing emergence of CDI^19^.

Some 16 articles included in this meta-analysis did not detect ST-1 (BI/NAP1/027) or ST-11 (RT 078) in Mainland China. According to our data, ST-37 (RT 017) strains were predominant in Mainland China. This result is in agreement with previous research results showing that ST-37 (RT 017) was the dominant type in Mainland China^36^,^37^. In contrast, one study showed that ST-37 (RT 017) strains were rare among healthy individuals^38^. However, we also learned from scattered reports that highly virulent strains, such as RT 078 and RT 027, have been isolated in Mainland China. In 2008, Cheng, V. C. *et al*. detected the RT 027 strain of *C. difficile* for the first time, and Huang, H. H. *et al*. first reported the discovery of RT 078 in Mainland China in 2011^39^,^40^. However, the epidemic strains in Mainland China are not the highly virulent type of *C. difficile*, which may be the reason why CDI has not had an outbreak in our country in recent years. Additionally, the number of reports about toxin A-negative and toxin B-positive strains has gradually increased in recent years^41^. Many studies in Mainland China have found the same situation^42^. In Asia, the numbers of toxin A-negative and toxin B-positive strains are significantly higher than that in European and American countries, which may also explain why the prevalent strain in Asia is RT 017^26^,^43^ and not RT 027. Nevertheless, rational tests and multi-centre or national-level surveillance for CDI, particularly for RT 027, should be introduced to provide essential data and guide future clinical practice^44^. RT 027 *C. difficile* isolates were detected in Beijing in 2012 and 2013, following the first discovery in Hong Kong^39^,^44^. Thus, it is time to stop neglecting CDI in Mainland China, and this problem should receive greater awareness.

CDI shows a close relationship with AAD, as described above. A number of international studies reported on the wide use of broad-spectrum antimicrobials, particularly the third generation of cephalosporin, the fluoroquinolone drugs and the clindamycin drugs, which subsequently increased the resistance to these drugs. Meanwhile, the balance of intestinal flora is destroyed following antibiotic treatment, which serves as a risk factor for *C. difficile*-associated diarrhoea (CDAD)^45^,^46^,^47^,^48^. The resistance rates to ciprofloxacin and cefoxitin were almost 100% in this study, which is consistent with these previous reports. According to the study by Pituch H *et al*., the rate of resistance to moxifloxacin was 38.5–40.1%, which is similar to that identified in this study (39.0%; 95% CI = 27.9–50.1%)^49^. Additionally, Ilchmann, C. and Oka, K. *et al*. found that the rates of resistance to clindamycin and erythromycin were more than 80%^50^,^51^, which is also similar to the results of our study ((81.7%; 95% CI = 76.1–87.3%); (80.2%; 95% CI = 73.5–86.9%), respectively). In recent years, a foreign survey reported the appearance of metronidazole- and/or vancomycin-resistant strains^52^. For example, Martin H *et al*. discovered that the rate of drug resistance to metronidazole and vancomycin was 1.8% and 0.4% respectively^53^. However, the articles included in this study did not identify metronidazole- and/or vancomycin-resistant strains. Therefore, the resistance profiles of *C. difficile* to these two drugs differ between Mainland China and other countries. In Mainland China, only heteroresistance to metronidazole was reported in Shanghai^54^, and this type of resistance only appears in the first generation of cultured strains and will disappear after several generations^55^. However, the epidemic ST-37 strain in Mainland China presented the highest resistance rate to many antibiotics, particularly clindamycin, tetracycline, and moxifloxacin, and all ST-37 isolates exhibited multi-drug resistance^56^. Some studies also indicated that the recurrence rate has increased in recent years following the use of metronidazole and/or vancomycin^57^. For example, the recurrence rates following metronidazole and vancomycin were reported at 25% and 60%, respectively^46^.

We realize that there are additional limitations of this study. First, we included few articles about *C. difficile* in Mainland China, particularly those reporting on the incidence and antibiotic resistance. Second, there is no standard test for detecting toxigenic *C. difficile*. Enzyme immunoassays and PCR were widely used to detect toxigenic *C. difficile*, even though the positive detection rate of toxigenic *C. difficile* was not statistically significant^58^. Third, the methods used to detect antibiotic resistance differed between studies. The agar dilution method and E-test were most commonly adopted in these articles. Moreover, the selection criteria for Topic 1 have disadvantages as well, as these criteria do not separate diarrhoeal patients into high-risk groups and low-risk groups, such as AAD and non-AAD. Moreover, studies published in local journals, which were not indexed in the electronic databases, might have been missed in this meta-analysis. Last, significant heterogeneity was observed between studies. For example, the incidence of toxigenic *C. difficile* among diarrhoea patients varied in different subgroups, such as outpatients and inpatients, ordinary inpatients and ICU patients. However, we did not obtain sufficient information about these subgroups for further analysis. Therefore, the prevalence and antibiotic resistance of *C. difficile* in Mainland China requires further study.

Despite the limitations listed above, there are some noteworthy findings of this study. First, the incidence of toxigenic *C. difficile* among diarrhoea patients in Mainland China was 14% (95% CI = 12–16%) (n/N = 2,132/15,313), and this rate may be slightly higher than the actual status because some patients included in Topic 1 had additional risk factors. Second, although RT 014/020 were the most common RT strains in Western Australia^8^, RT 017 (ST-37) was the major epidemic strain in Mainland China. The existence of RT 027 (ST-1) and RT 078 (ST-11) in Mainland China was only reported in a few studies, whereas these are the dominant strains in outbreaks in Europe and North America. Finally, the rates of resistance to erythromycin and clindamycin were greater than 80%, and the rates of resistance to ciprofloxacin and cefoxitin almost reached 100%. These results in Mainland China are consistent with other reports on *C. difficile* drug resistance in other countries. Most importantly, Mainland China has not detected the metronidazole- and vancomycin-resistant strains of *C. difficile* to date. Together, the information provided in this study will help guide rational drug use in the clinic and enhance the awareness of C. *difficile* infection and the epidemiological characteristics of these infections in Mainland China.

## Methods

### Search strategy

We searched the NCBI PubMed, Web of Science, Science Director, OVID, China National Knowledge Infrastructure (CNKI), Wanfang (Chinese) and Weipu (Chinese) databases to identify research studies that described *C. difficile* infections in Mainland China. The following keywords were used in the searches: “*C. difficile*”, “prevalence”, “incidence”, “molecular epidemiology”, “in Mainland China” and “antibiotic resistance.” The search was limited to publications from 2010 to 2016. The same strategies were used for each database. We placed no language restrictions on the searches or search results.

### Selection criteria

Two investigators (Chenjie Tang and Lunbiao Cui) independently reviewed the potentially appropriate studies to determine whether they met the predetermined eligibility criteria. Disagreements between the reviewers were resolved by consulting the other authors. Studies obtained from the literature search were checked by titles and abstracts. The titles and abstracts of the potential references were carefully scanned to exclude irrelevant articles. The remaining articles were evaluated to identify research that contained the relevant information, and the full texts were then reviewed in depth. In addition, the inclusion and exclusion criteria were established by the investigators prior to the literature review, and we also estimated and scored the relevance and quality of the references according to JBI (Joanna Briggs Institute). The inclusion criteria were as follows: Topic 1: *C. difficile* and its toxins were detected in stool samples from diarrhoea patients; Topic 2: *C. difficile* was detected by MLST or RT methods; and Topic 3: AST was performed on *C. difficile*. Moreover, studies were only included if they constituted original works and if the samples in these studies came from Mainland China. Only studies published within the last 6 years were included. Studies were excluded if they met the following conditions: (1) review or case report; (2) the data were not from 2010–2016; (3) studies with less than 5 samples; (4) samples from different studies were repeated; (5) the samples did not come from Mainland China; (6) the studies were repeated in different languages; (7) the information was not complete; and (8) the samples did not come from the diarrhoea patients included in Topic 1. Figure 1 shows the flowchart of the procedure used to select the articles.

### Data extraction

All data were extracted by two independent investigators. Disagreements in data extraction were resolved by reaching a consensus in accordance with the original study and the other authors’ opinions. The following relevant data were extracted in three predefined tables. Details about the data extraction can be found in the Supplementary Tables 1, 2 and 3.

### Statistical analysis

Microsoft Excel (version 12.0) and Stata (version 12.0) were used in this meta-analysis. Based on the possibility of significant heterogeneity, we used the Q-statistic (p < 0.05 was considered to indicate statistically significant heterogeneity) to estimate the heterogeneity between studies. A random effect model (REM) or fixed effect model (FEM) was chosen for the meta-analysis according to the p value. We calculated the incidence of toxigenic *C. difficile* among diarrhoea patients in the provinces of Mainland China, as well as the molecular epidemiology and antibiotic resistance using 95% confidence intervals (CIs) and a suitable model. Data manipulation and statistical analyses were performed using Stata 12.0(StataCorp. 2011. Stata Statistical Software: Release 12. College Station, TX: StataCorp LP.).

## Additional Information

**How to cite this article**: Tang, C. *et al*. The incidence and drug resistance of *Clostridium difficile* infection in Mainland China: a systematic review and meta-analysis. *Sci. Rep.* **6**, 37865; doi: 10.1038/srep37865 (2016).

**Publisher's note:** Springer Nature remains neutral with regard to jurisdictional claims in published maps and institutional affiliations.

## Supplementary Material

Supplementary Table 1

Supplementary Table 2

Supplementary Table 3

## Figures and Tables

**Table 1:** The incidence of CDI in Mainland China.

Province	Incidence of CDI (95% CI) (%)	n/N	Weight	References
Anhui	19(12,26)	24/126	5.04	59
Beijing	13(11,14)	230/1,791	8.88	60, 61, 62, 63, 64, 65
Gansu	13(8,17)	26/206	6.78	66
Guangdong	15(14,16)	789/5,289	9.12	24, 67, 68, 69, 70, 71
Hebei	19(15,24)	66/340	7.04	23, 27
Henan	3(−1,8)	2/60	6.77	72
Hubei	23(20,26)	170/742	7.96	73, 74, 75
Hunan	16(14,18)	188/1,202	8.62	36, 76, 77, 78
Jiangsu	16(12,20)	42/262	6.85	79, 80
Ningxia	4(1,6)	9/233	8.35	81
Shanghai	13(11,15)	138/1,053	8.63	82, 83, 84
Sichuan	17(13,22)	48/279	6.86	85, 86
Zhejiang	11(10,12)	400/3,730	9.11	19, 58, 87, 88, 89, 90, 91
Overall	14(12,16)	2,132/15,313	100	39

n: number of toxigenic *C. difficile* species.

N: total number of samples from the studies.

**Table 2:** The main MLST typing of *C. difficile* reported in Mainland China.

MLST	Molecular epidemiology of *C. difficile* (95% CI) (%)	Chi-squared	P	Model	n/N	References
ST-1	0	—	—	—	0/407	19, 23, 24, 27, 82, 87
ST-2	0.086(0.05–0.118)	1.41	0.494	FEM	26/288	24, 38, 78
ST-3	0.181(0.083–0.278)	8.36	0.039	REM	67/295	24, 38, 78, 92
ST-11	0	—	—	—	0/280	24, 27, 82, 87
ST-26	0.123(0.042–0.204)	0.5	0.479	FEM	8/62	24, 78
ST-35	0.136(0.063–0.210)	16.00	0.003	REM	64/455	36, 38, 78, 87, 92
ST-37	0.172(0.122–0.221)	43.77	0	REM	152/913	19, 24, 36, 37, 38, 42, 77, 78, 82, 87, 92, 93, 94
ST-39	0.159(0.068–0.250)	0.17	0.68	FEM	10/62	24, 78
ST-54	0.167(0.098–0.237)	50.99	0	REM	146/711	24, 36, 38, 42, 77, 78, 82, 87, 93

MLST: Multiple Locus Sequence Typing; n: number of events; N: total number of samples from the studies. —: The data were not applied to the statistical calculation.

**Table 3:** The main ribotyping of *C. difficile* in Mainland China.

RT	Epidemiology of *C. difficile* (95% CI) (%)	Chi-squared	P	Model	n/N	References
RT 001	0.114(0.036–0.191)	24.81	0	REM	42/298	19, 42, 77, 90, 93
RT 002	0.157(−0.105–0.419)	12.51	0	REM	14/103	77, 90
RT 006	0.55(0.396–0.704)	—	—	—	22/40	90
RT 012	0.167(0.098–0.237)	50.99	0	REM	146/711	24, 36, 38, 42, 77, 78, 82, 87, 93
RT 014	0.127(0.068–0.186)	0.56	0.454	FEM	16/122	19, 90
RT 017	0.172(0.122–0.221)	43.77	0	REM	152/913	19, 24, 36, 37, 38, 42, 77, 78, 82, 87, 92, 93, 94
RT 027	0	—	—	—	0/407	19, 23, 24, 27, 82, 87
RT 046	0.136(0.063–0.210)	16.00	0.003	REM	64/455	36, 38, 78, 87, 92
RT 078	0	—	—	—	0/280	24, 27, 82, 87

n: number of events; N: total number of samples from the studies.

—: The data were not applied to the statistical calculation.

**Table 4:** The antibiotic resistance of *C. difficile* in Mainland China in the past 6 years.

Antimicrobial agents	Drug resistance (95%CI) (%)	Chi-squared	P	I-squared	Model	n/N	References
Metronidazole	0	—	—	—	—	0/960	23, 24, 25, 26, 27, 28, 29, 40, 54, 94
Vancomycin	0	—	—	—	—	0/960	23, 24, 25, 26, 27, 28, 29, 40, 54, 94
Tigecycline	0	—	—	—	—	0/41	27, 94
Piperacillin/Tazobactam	0	—	—	—	—	0/288	24, 40, 54
Erythromycin	80.2(73.5–86.9)	8.26	0.041	63.70%	REM	340/433	25, 26, 40, 54, 94
Clindamycin	81.7(76.1–87.3)	13.08	0.023	61.80%	REM	476/581	24, 25, 26, 27, 29, 40, 54, 94
Tetracycline	46.8(36.7–56.9)	15.9	0.001	81.10%	REM	231/498	26, 29, 40, 54
Moxifloxacin	39.0(27.9–50.1)	38.79	0	84.50%	REM	247/549	24, 25, 26, 29, 40, 54, 94
Ciprofloxacin	98.3(96.9–99.7)	0	—	—	FEM	688/694	28, 29, 40, 54
Fusidic acid	16.8(5.4–28.2)	21.06	0	90.50%	REM	72/404	26, 40, 54
Rifampicin	18.3(7.2–29.4)	59.61	0	93.30%	REM	89/527	25, 26, 29, 40, 54, 94
Rifaximin	22.1(17.1–27.0)	2.48	0.115	59.70%	FEM	60/600	28, 40, 54
Meropenem	8.8(−8–25.6)	6.25	0.012	84.00%	REM	11/388	24, 27, 28
Levofloxacin	60.2(44.4–75.9)	97.42	0	94.9%	REM	436/779	26, 27, 28, 40, 54, 94

n: number of drug resistant strains; N: total number of *C. difficile* strains from the included studies.

“—” The data were not applied to the statistical calculation.
